# Prognostic Role of the Ubiquitin Proteasome System in Clear Cell Renal Cell Carcinoma: A Bioinformatic Perspective

**DOI:** 10.7150/jca.53760

**Published:** 2021-05-13

**Authors:** Hongda Guo, Yan Li, Yaxiao Liu, Lipeng Chen, Zhengdong Gao, Lekai Zhang, Nan Zhou, Hu Guo, Benkang Shi

**Affiliations:** 1 Department of Urology, Qilu Hospital of Shandong University, 107 Wenhuaxi Road, Jinan, 250012, China.; 2 Key Laboratory of Urinary Precision Diagnosis and Treatment in Universities of Shandong, Jinan, P.R. China.

**Keywords:** the ubiquitin proteasome system (UPS), clear cell renal cell carcinoma (ccRCC), prognosis, bioinformatics

## Abstract

**Background:** Clear cell renal cell carcinoma (ccRCC) is a common malignant tumor of the urinary system. The ubiquitin proteasome system (UPS) plays an important role in the generation, metabolism and survival of tumor. We are aimed to make a comprehensive exploration of the UPS's role in ccRCC with bioinformatic tools, which may contribute to the understanding of UPS in ccRCC, and give insight for further research.

**Methods:** The UPS-related genes (UPSs) were collected by an integrative approach. The expression and clinical data were downloaded from TCGA database. R soft was used to perform the differentially expressed UPSs analysis, functional enrichment analysis. We also estimated prognostic value of each UPS with the help of GEPIA database. Two predicting models were constructed with the differentially expressed UPSs and prognosis-related genes, respectively. The correlations of risk score with clinical characteristics were also evaluated. Data of GSE29609 cohort were obtained from GEO database to validate the prognostic models.

**Results:** We finally identified 91 differentially expressed UPSs, 48 prognosis related genes among them, and constructed a prognostic model with 18 UPSs successfully, the AUC was 0.760. With the help of GEPIA, we found 391 prognosis-related UPSs, accounting for 57.84% of all UPSs. Another prognostic model was constructed with 28 prognosis-related genes of them, and with a better AUC of 0.825. Additionally, our models can also stratify patients into high and low risk groups accurately in GSE29609 cohort. Similar prognostic values of our models were observed in the validated GSE29609 cohort.

**Conclusions:** UPS is dysregulated in ccRCC. UPS related genes have significant prognostic value in ccRCC. Models constructed with UPSs are effective and applicable. An abnormal ubiquitin proteasome system should play an important role in ccRCC and be worthy of further study.

## Introduction

Renal cell carcinoma (RCC) is a type of malignant tumor originating from the renal tubular epithelium and accounts for about 3-5% of all adult malignancies 1. Clear cell renal cell carcinoma (ccRCC) is the most common histological subtype and accounts for approximately 75% of RCC 2. Surgical resection is the main treatment for ccRCC, but ccRCC is likely to recur and threatens patient's life 3-5. Therefore, the exploration of new molecular targets in ccRCC is essential as well as valuable for the development of more accurate diagnosis and more effective treatment. The ubiquitin proteasome system (UPS) seems a promising candidate 6.

The UPS is a specialized proteolysis system which is responsible for protein degradation and plays an essential role in the regulation of abundant biological processes 7, 8. It consists of a series of key components: ubiquitin, ubiquitin activating enzymes (E1s), ubiquitin conjugating enzymes (E2s), ubiquitin ligases (E3s), deubiquitinating enzymes (DUBs), and the proteasome 9. The UPS controls the degradation of more than 80% of cellular proteins and plays an indispensable role in a wide range of biological processes including cell proliferation, development, immune responses, and a variety of human diseases including tumor 10, 11. Dysregulation of UPS is closely related to tumor and tumor cells are highly dependent on a functional UPS 12. Considering the current evidence indicates aberrancies in the UPS pathway play an important role in cancer, development of drugs targeting different components of the UPS has been proposed as a promising therapeutic strategy 13-17. An inspiring example is the success of proteasome inhibitors used in multiple myeloma treatment 18, 19.

Previous researches suggest ccRCC is dominated by inactivating mutations in Von-Hippel Lindau (VHL). The protein encoded by VHL is involved in assembling a protein complex possessing ubiquitin ligase E3 activity. It is responsible for the ubiquitination and degradation of hypoxia-inducible-factor (HIF), which plays a central role in the control of gene expression by oxygen 20. Besides VHL, there are also some other UPS genes have been reported to be involved in ccRCC such as USP2 21, USP44 22, CUL5 23, SPOP 24 and UHRF1 25. Additionally, UPS was also found to be involved in papillary renal cell carcinoma 26. However, there are few researches focusing on exploring the potential of the whole ubiquitin proteasome system in ccRCC. In this study, we are aimed to make a comprehensive exploration of the ubiquitin proteasome system's role in ccRCC, mainly focus on the prognostic value of UPS-related genes (UPSs) with the assistance of bioinformatic tools.

## Materials and Methods

### Collection of UPSs

We collected and confirmed the genes encoding the ubiquitin proteasome system (UPS) by an integrative approach. Coding genes of ubiquitin were obtained from the Uniport database, while the gene lists of ubiquitin activating enzymes (E1s) 27, ubiquitin conjugating enzymes (E2s) 28 and deubiquitinating enzymes (DUBs) 29 were collected from the reliable published literatures. There are several online databases for ubiquitin ligases (E3s), among them we select the database named UbiNet, for it have collected abundant members of E3 ligases in human, about 500 genes 30. As for the proteasome, which is a relatively conservative component of UPS, we get its genes from GSEA website, by downloading the gene set named KEGG_PROTEASOME 31, 32. Finally, we get the UPS-related gene set containing 676 UPSs in total. Details of gene set are shown in **Table S1.**

### Samples and data retrieval

We acquired both the FPKM-standardized RNA-seq data and the clinical information from the KIRC cohort in the TCGA database. All patients in TCGA-KIRC cohort are involved into our study when doing expression related analyses, while patients with unknow clinical features were excluded in Cox regression analysis. Finally, we extracted an expression matrix of 670 UPSs in 72 normal samples and 539 tumor samples, and obtain a file containing complete clinical information without missing values in 248 patients.

### Identification of differentially expressed UPSs

R software (version 4.0.2) and “limma” package were used to make the analysis of differentially expressed UPSs. We set a false discovery rate (FDR) < 0.05 and a | log2 fold change (FC) | > 1 as screening criteria to obtain the differentially expressed UPSs. We also visualized them by the heatmap and boxplot.

### Enrichment analysis of differentially expressed UPSs

To have a better understanding of differentially expressed UPSs in ccRCC, we carried out a series of gene functional enrichment analyses to determine the major biological attributes, including the gene ontology (GO) and Kyoto Encyclopedia of Genes and Genomes (KEGG) analyses. The “GOplot” package was employed to visualize the enrichment terms 33.

### Construction of prognostic model (Model 1) with differentially expressed UPSs

We obtained 48 prognostic related UPSs in ccRCC by univariate Cox regression analysis, and these prognosis-related genes were used to construct a prognostic model by multivariate Cox regression with the step function in R. This model was used to calculate the risk score for every ccRCC patient, and the patients were divided into the low-risk group and high-risk group. Survival analysis was made by Kaplan-Meier method. Cox regression analysis was performed to demonstrate whether the UPS related risk score was an independent prognostic factor in ccRCC patients. Heatmap was employed to visualize the expression pattern between two groups. We also made GSEA analysis in GSEA software (version 4.0.1) to analyze which pathways genes are primarily enriched in high- and low-risk groups 32.

### Exploration of all 676 UPSs in GEPIA

GEPIA, which is an interactive web server developed for exploring the RNA sequencing expression data from the TCGA and the GTEx projects, was used in this part 34. The excellent function of patient survival analysis provided by GEPIA was used to estimate each prognostic value of all 676 UPSs. With the log-rank p < 0.05 and | log2 hazard rate (HR) | > 1, we got 69 prognosis-related genes with outstanding prognostic value. The correlations of gene expression and tumor stage were also explored with the stage plot function of GEPIA.

### Construction of prognostic model (Model 2) with 28 prognosis related UPSs

Considering the extraordinary significance of 69 prognosis-related genes, we constructed another prognostic model with these genes by multivariate Cox regression with the step function in R. 28 UPSs were involved in the Model 2. We also got a risk score formula and made survival analysis between two groups separated by calculated risk score value. Cox regression analysis was performed to support this risk score as an independent prognostic factor.

### Estimation and external validation of Model 1 and Model 2

Finally, we estimated these two models by drawing receiver operating characteristic (ROC) curve with the “survivalROC” package. Correlation of risk score with clinical characteristics were also evaluated. Additionally, we downloaded data of GSE29609 from GEO database, GSE29609 provided data of whole genome expression of 39 clear-cell renal cell carcinomas patient as well as survival state and survival time 35. This 39 ccRCC patients were used for external validation and their risk scores were calculated with the formulas of Model 1 and Model 2, respectively. Kaplan-Meier curve was plotted with R software.

### Statistical Analysis

Statistical analyses were performed with R software (Version 4.0.2). The Wilcox signed-rank test was used to compare the expression levels of differentially expressed UPSs between cancer tissues and normal tissues. Cox regression analyses were employed to filter genes to build risk score models. Differences between survival curves generated by the Kaplan-Meier method were defined by log-rank tests. Receiver operating characteristic (ROC) curve analysis was performed with the “survivalROC” package. The Wilcoxon rank sum test was used in estimating the correlations of risk score with clinical characteristics. All statistical tests were bilateral, with p < 0.05 being statistically significant.

## Results

### Differentially expressed UPSs in tumor samples comparing with normal samples

**Figure 1:**
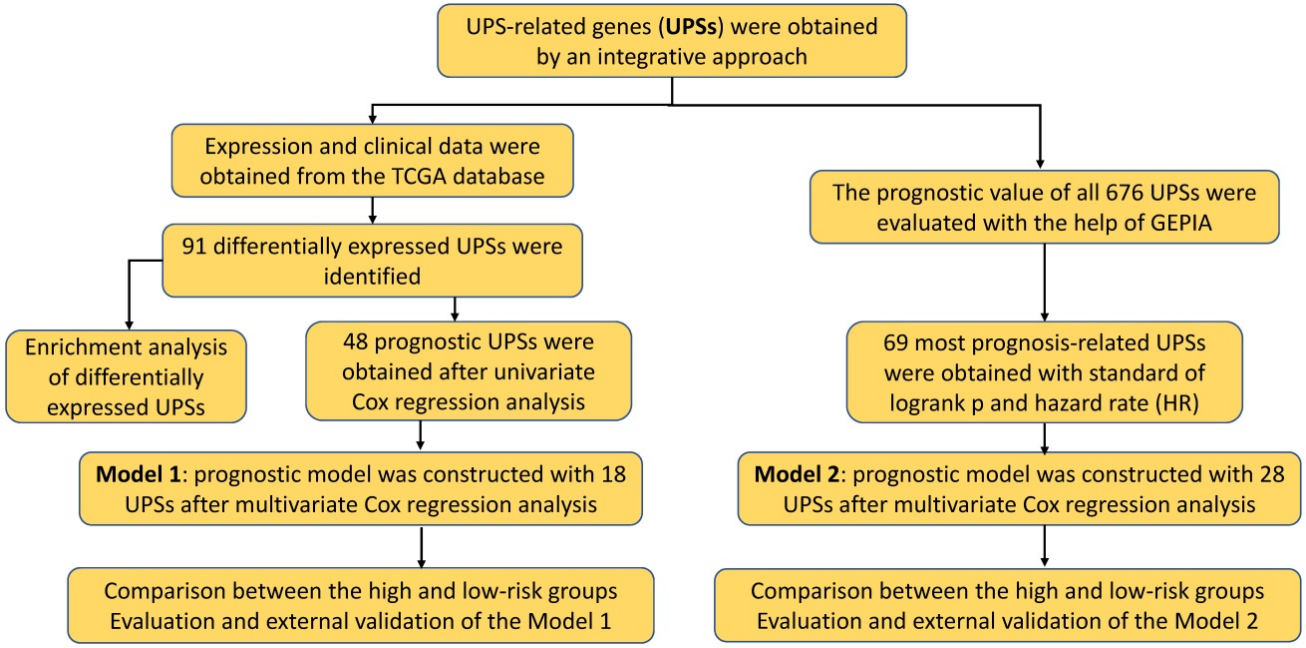
** Overview flowchart of this study.** Exploration of the prognostic role of UPS in ccRCC from a bioinformatic perspective.

**Figure 2:**
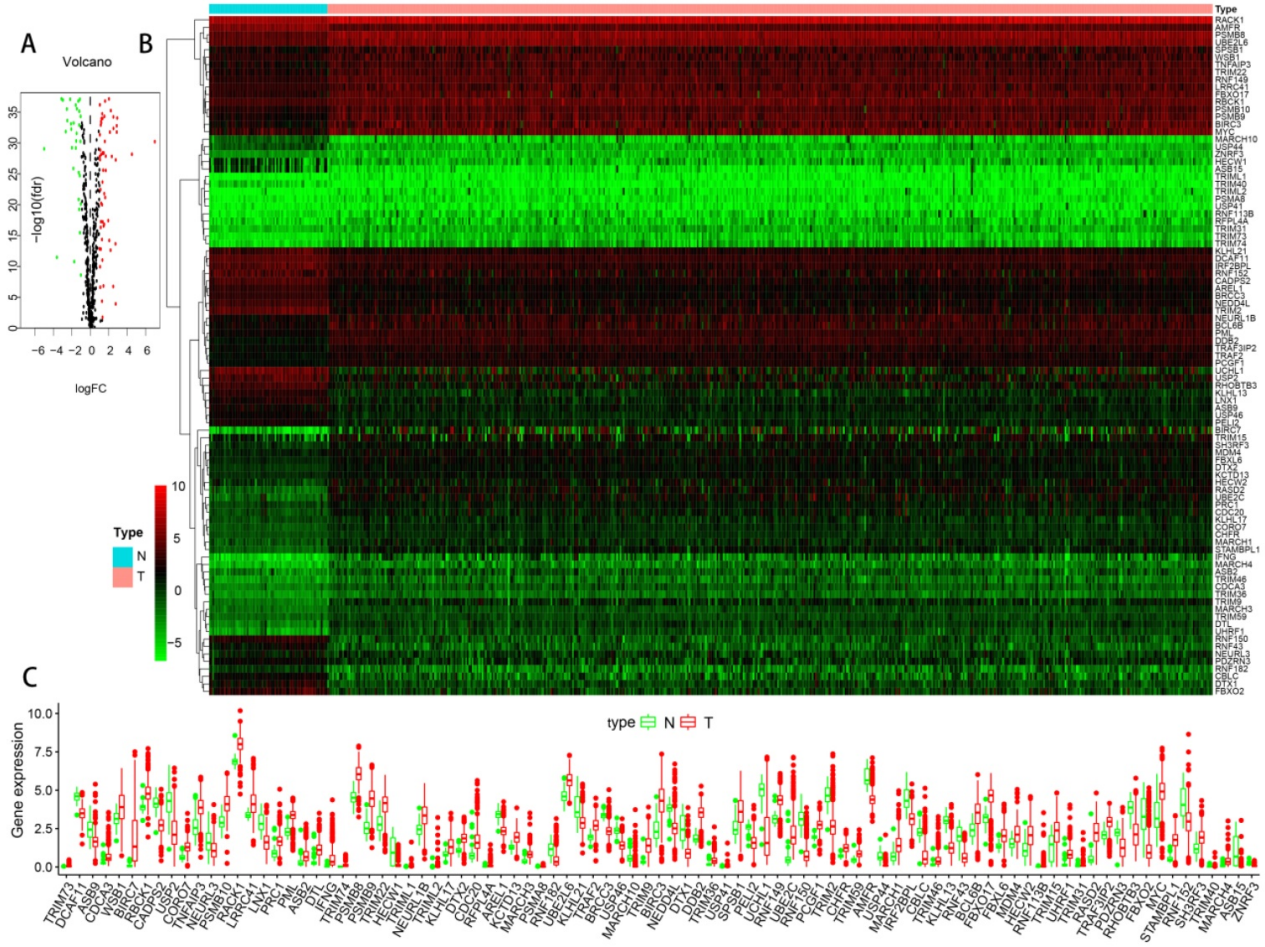
** The expression profiles of UPSs between tumor samples and normal samples in TCGA cohort of ccRCC.** (A) Volcano plot of 676 UPSs. The vertical axis indicates the -log10 False Discovery Rate (FDR), and the horizontal axis indicates the log2 fold change (FC). The red dots and the green dots represent up- and down-regulated genes, respectively (P-value < 0.05 and |log2(FC)| > 1). (B) Heatmap of 91 differentially expressed UPSs. Red and green indicate higher expression and lower expression, respectively. (C) Box plot of the expression of 91 differentially expressed UPSs between tumor and normal tissues, tumor in red and normal in green.

The flowchart of our research was showed in **Figure 1**, detailed clinical parameters of patients were shown in **Table 1.** The expression values of UPSs were extracted from normal and tumor samples. With FDR < 0.05 and | log2 FC | > 1 as the screening criteria, 91 differentially expressed UPSs were obtained. Compared with normal samples, there were 32 downregulated UPSs and 59 upregulated UPSs in tumor samples, details are shown in **Table 2.** Many of them are not clearly elucidated and waiting for more extensively studies. We also visualized the expression pattern of these genes below (**Figure 2**). It is not difficult to find that there are more upregulated genes than downregulated genes in tumor samples. These findings may show that tumor needs a relatively high activity of UPS to survive and imply these UPSs are potential novel targets for treatment.

### Functional enrichment of the differentially expressed UPSs

**Figure 3:**
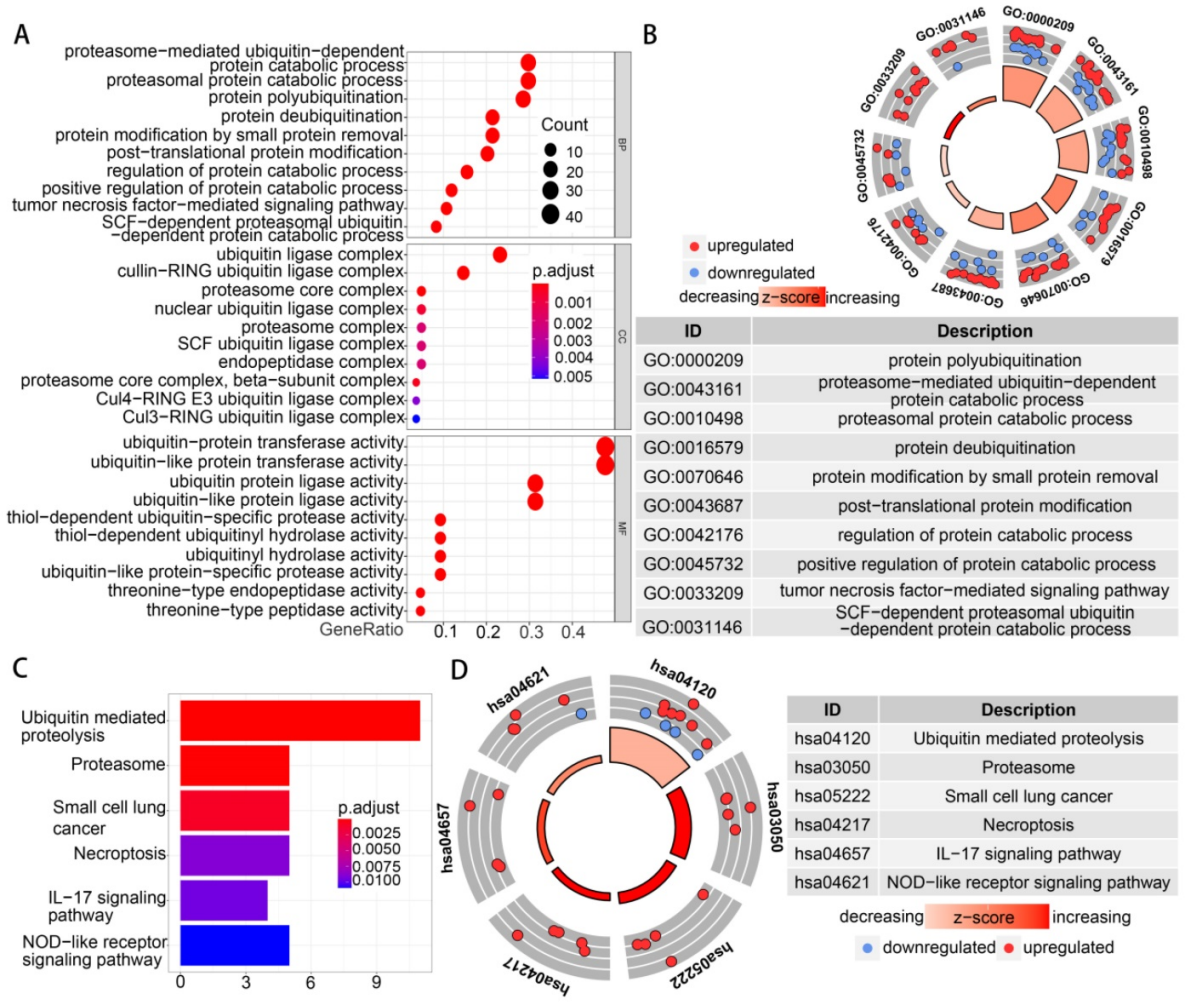
** Functional enrichment of the differentially expressed UPSs.** (A) The top 30 significant terms of GO function enrichment. BP biological process, CC cellular component, MF molecular function. (B) The GO circle shows the scatter map of the log FC of the specified gene. (C) The terms of KEGG analysis with statistical significance. (D) The KEGG circle shows the scatter map of the log FC of the specified gene. The higher the z-score value indicated, the higher expression of the enriched pathway.

Functional enrichment analysis was performed with 91 differentially expressed UPSs. The GO term functional enrichment and the KEGG pathway enrichment analysis of these genes were summarized in **Figure 3**. In the GO terms in biological processes, differentially expressed UPSs were mainly enriched in protein polyubiquitination, proteasome-mediated ubiquitin-dependent protein catabolic process, proteasomal protein catabolic process, protein deubiquitination, protein modification by small protein removal. In the cellular components, differentially expressed UPSs were mainly enriched in ubiquitin ligase complex, cullin-RING ubiquitin ligase complex, proteasome core complex, proteasome core complex (beta-subunit complex), nuclear ubiquitin ligase complex. The top enriched GO terms in the molecular functions were ubiquitin-protein transferase activity, ubiquitin-like protein transferase activity, ubiquitin protein ligase activity, ubiquitin-like protein ligase activity, thiol-dependent ubiquitin-specific protease activity. In the KEGG pathway enrichment analysis, the UPSs were mainly enriched in Ubiquitin mediated proteolysis, Proteasome, Small cell lung cancer, Necroptosis, IL-17 signaling pathway, NOD-like receptor signaling pathway. The z scores of these pathways were > 0, indicating that the UPSs were upregulated in these pathways, which indicated a relatively high activity of these pathways in tumor samples. More details are shown in **Table S2.**

### Model 1: construction of a prognostic signature with differentially expressed UPSs

**Figure 4:**
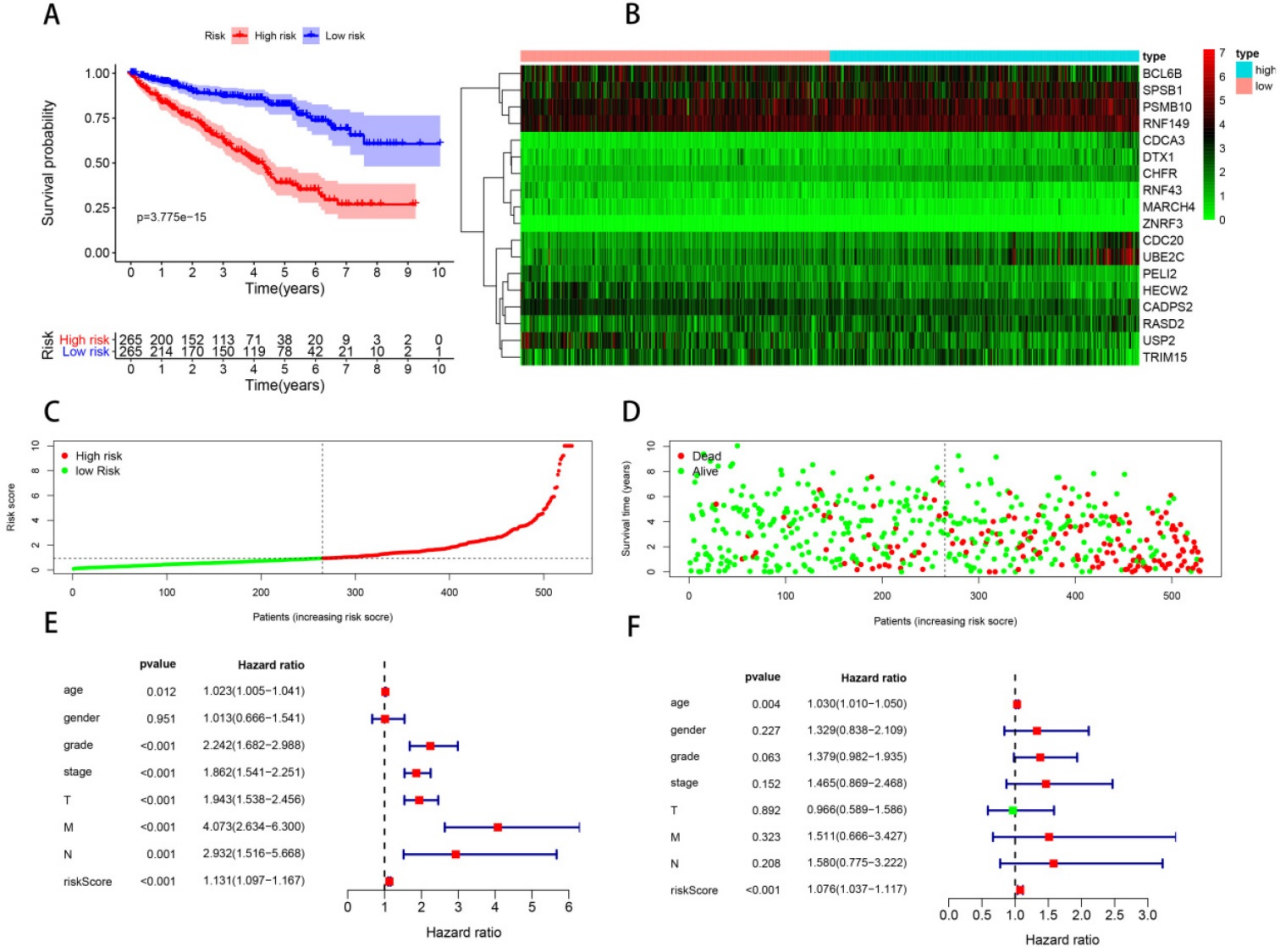
** Model 1: prognostic signature constructed with 18 differentially expressed UPSs.** (A) Kaplan-Meier curves of OS in the high- and low-risk groups stratified by the median risk score. (B) Heatmap of the expression profile of the model genes in two groups. (C) Distribution of the risk scores of ccRCC patients. (D) Survival status of patients in different groups, red dots denote patients that are dead, and green dots denote patients that are alive. (E) A forest plot of univariate Cox regression analysis in the cohorts. (F) A forest plot of multivariate Cox regression analysis in the cohorts.

In order to construct a prognostic signature, we first made univariate Cox regression analysis of survival with 91 differentially expressed UPSs, and 48 UPSs were found to be associated with the prognosis. In other words, more than half of the differentially expressed UPSs, up to 52.75%, have statistical significance with ccRCC patients' survival, which have surprised us a lot. After multivariate Cox regression analysis, 18 UPSs were identified and they were used to construct a prognostic signature. We named this prognostic signature “**Model 1**”. The risk score formula, shown in **Table 3**, was used to calculate the risk score of each patient, and patients were classified into the high-risk group (n = 265) and the low-risk group (n = 265) comparing with the median risk score. The different expression pattern of these 18 UPSs in two groups was shown by a heatmap (**Figure 4B**). It is obvious that the expression pattern proves essentially different in two groups. Particularly, we can easily find that the CDC20 and UBE2C are highly expressed in the high-risk group, while in the low-risk group, USP2 is highly expressed.

Kaplan-Meier analysis was performed to evaluate the overall survival (OS) in the two groups. As is shown, a significant difference in the survival rate between the high- and low-risk groups was observed (P = 3.775e-15, **Figure 4A**). Patients in the high-risk group had a worse OS than those in the low-risk group. The survival rates of 1, 3, 5 years in low-risk group are about 95.0%, 87.2%, 81.2%, while in the high-risk group, they are significantly reduced to only about 83.7%, 62.6%, 37.8%. We also visualized the relationship between risk score and patients' survival state. As is shown in the dot plot, there is an increasing number of patients died with the increase of risk score (**Figure 4D**). These results support the presume that the risk score accurately reflect the survival of patients. In order to determine whether the UPS-related signature for OS is an independent prognostic factor, univariate and multivariate Cox regression analyses were performed. As the results of univariate Cox regression analysis suggested, age, stage, grade, T stage, N stage, M stage and risk score were all significantly associated with OS in ccRCC patients. Multivariate Cox regression analysis showed that age and risk score were significantly associated with OS. These results support risk score as an independent prognostic factor in ccRCC patients.

**Figure 5:**
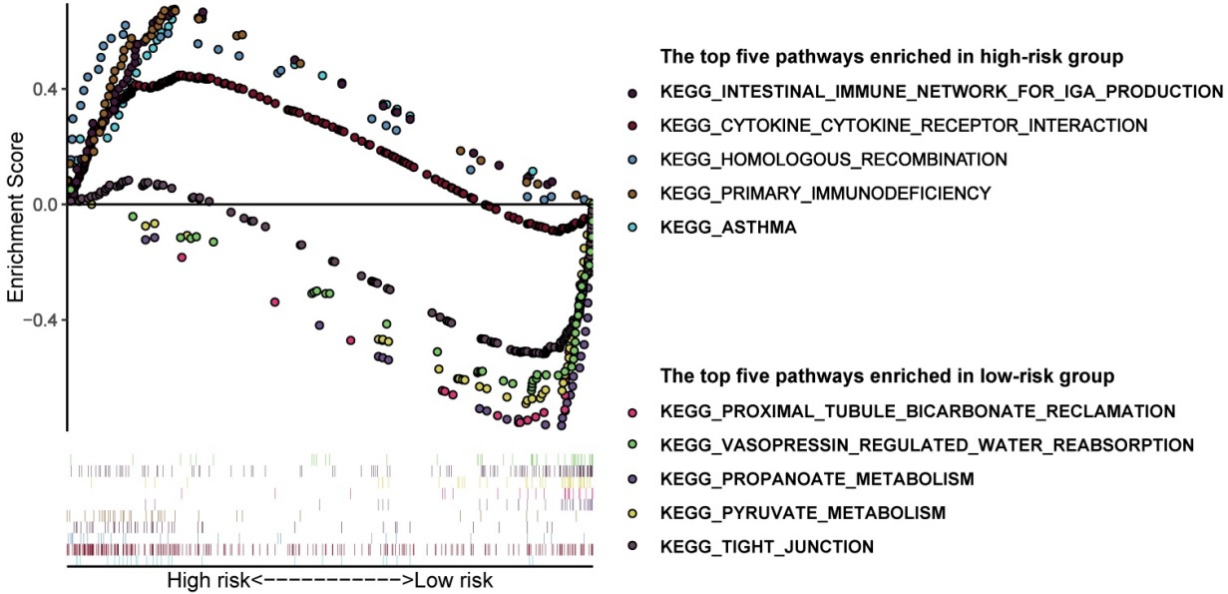
** Gene set enrichment analysis in different groups identified by Model 1.** The top 5 pathways enriched in the high-risk group and low-risk group, respectively.

In order to have a more comprehensive understanding of the difference between high and low-risk groups, we performed GSEA analysis. Results suggested that the pathways enriched in the two groups were really different (**Figure 5**). The following pathways were mostly enriched in the low-risk group: proximal tubule bicarbonate reclamation, vasopressin regulated water reabsorption, propanoate metabolism, pyruvate metabolism, tight junction. And in the high-risk group, the following pathways were mostly enriched: intestinal immune network for IgA production, cytokine-cytokine receptor interaction, homologous recombination, primary immunodeficiency, asthma. We can roughly conclude that the tumor tissues in low-risk group may have a better histologic differentiation and a lower malignancy phenotype, for its main enrichment pathways are more likely to occur in normal renal cells which may means the low-risk group tumor samples are more like normal tissues. On the contrary, the pathways enriched in the high-risk group are more related to immune response which may means the tumor tissues are worse differentiated and have a higher malignancy which have induced a relatively more violently immune response against the tumor in the body.

### Prognostic value of all UPSs

**Figure 6:**
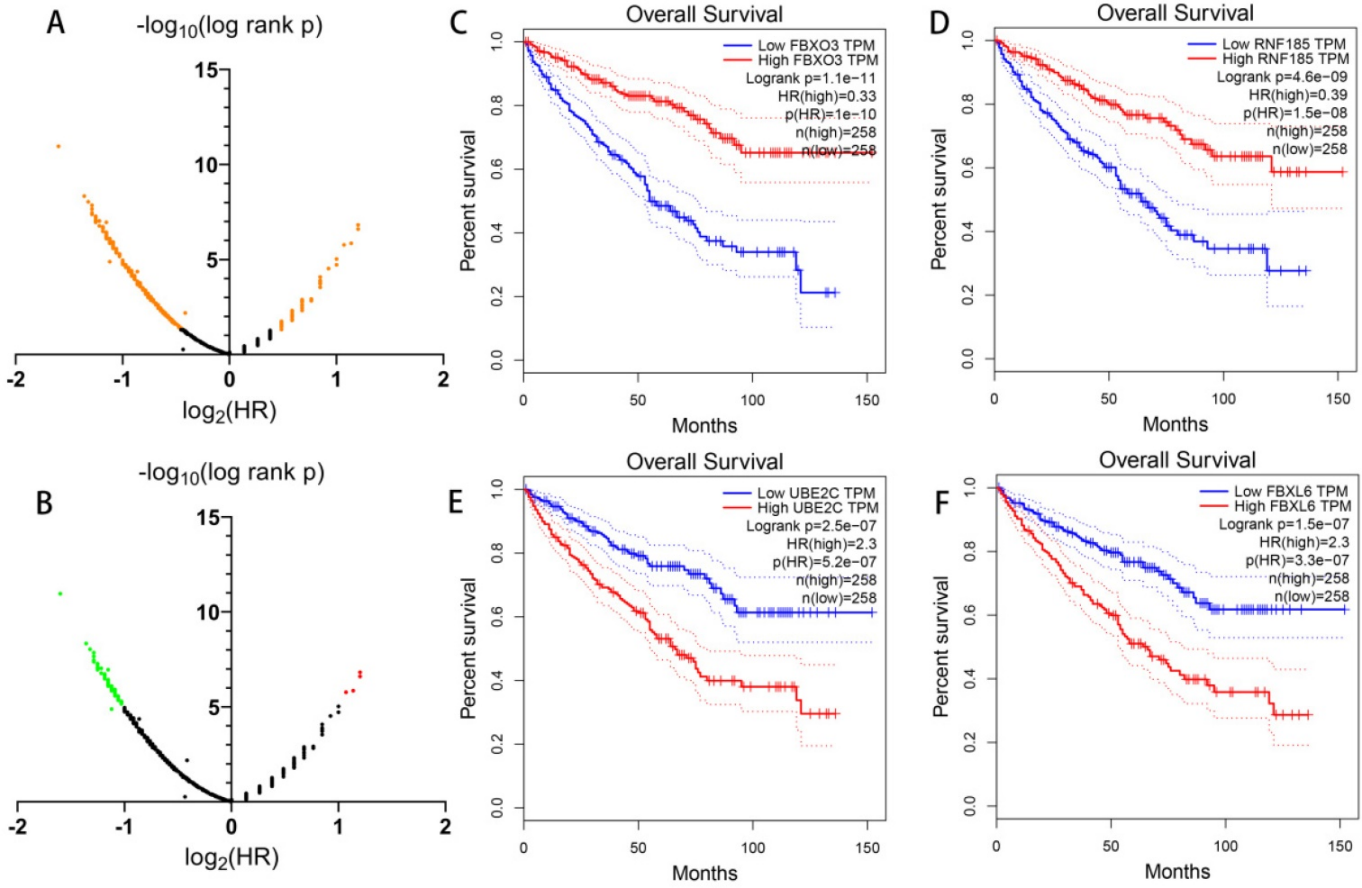
** Prognostic value of all UPS-related genes exploring with GEPIA.** (A) Dot plot to show the large proportion of UPSs with significant prognostic value, significant genes are shown in orange. (B) Dot plot to show the UPSs with outstanding prognostic value, red dots represent genes when log2 hazard rate (HR) > 1, green dots represent genes when log2 hazard rate (HR) < -1. (C-F) Kaplan-Meier curves of OS of representative genes with outstanding prognostic value.

Apart from constructing a prognostic signature with differentially expressed UPSs, in order to have a more comprehensive understanding of the UPSs' role in ccRCC, we also analyze the prognostic value of all 676 UPSs with the help of GEPIA (**Figure 6**). With log-rank p < 0.05, we found 391 prognosis-related UPSs in total among 676 UPSs, which means 57.84% UPSs had a significant relationship with the overall survival of ccRCC patients in TCGA cohort. Considering the fact that such a large proportion of UPSs had a significant correlation with the prognosis of ccRCC, we infer that the dysregulation of ubiquitin proteasome system should play a vital role in the occurrence and development of ccRCC.

**Figure 7:**
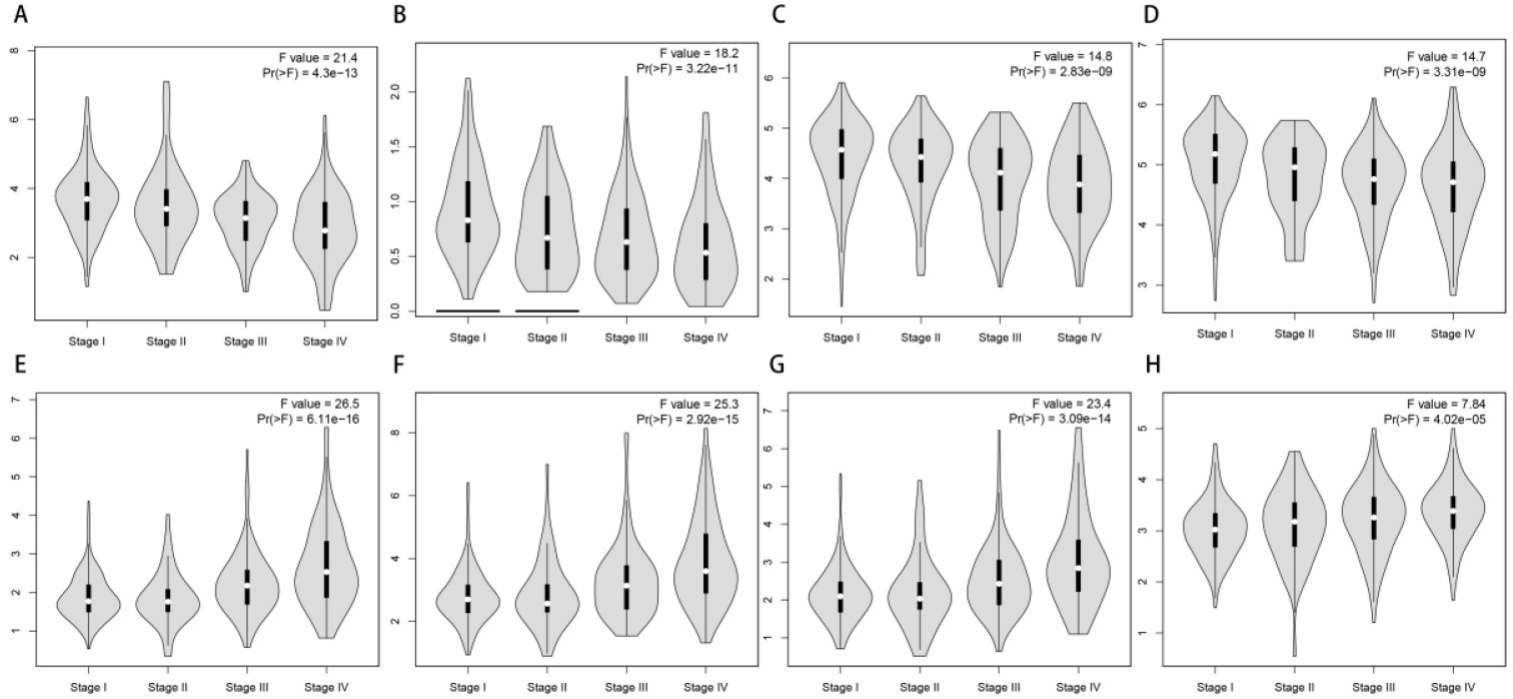
** Correlation of the expression of UPSs with tumor stages.** (A-D) 4 representative genes that have negative correlations with tumor stages. They are TRIM2 (A), OTUD7A (B), RCHY1 (C), DCAF11 (D). (E-H) 4 representative genes that have positive correlations with tumor stages. They are CDCA3 (E), UBE2C (F), CDC20 (G), FBXL6 (H).

With the convenience of GEPIA, we also made an exploration of the correlation of the expression of 69 most prognosis related genes and tumor stage. To our surprise, most of them have a significance relationship with tumor stage. If we see genes as “good genes” when high expression followed with better survival, and “bad genes” when high expression comes with worse survival. Then we can find there are 65 “good genes” among them, and most of 65 “good genes” are negatively correlate with tumor stage, while the only 4 “bad genes” (CDC20, CDCA3, FBXL6, UBE2C) are positively correlate with tumor stage (**Figure 7**).

### Model 2: construction of prognostic model with 28 prognosis-related UPSs

**Figure 8:**
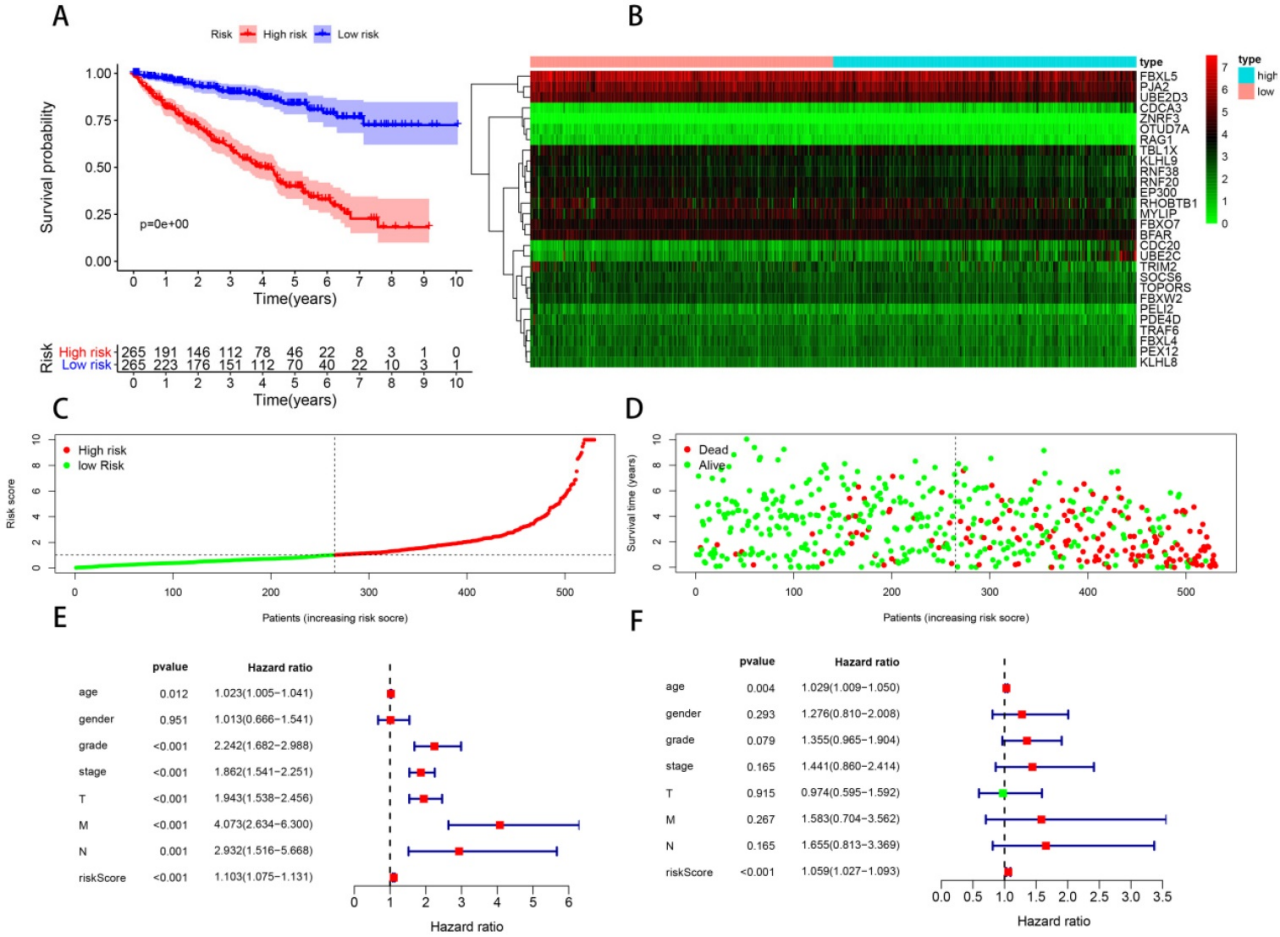
** Model 2: prognostic signature constructed with 28 prognosis related UPSs.** (A) Kaplan-Meier curves of OS in the high- and low-risk groups stratified by the median risk score. (B) Heatmap of the expression profile of the model genes in two groups. (C) Distribution of the risk scores of ccRCC patients. (D) Survival status of patients in different groups, red dots denote patients that are dead, and green dots denote patients that are alive. (E) A forest plot of univariate Cox regression analysis in the cohorts. (F) A forest plot of multivariate Cox regression analysis in the cohorts.

Making a prognostic model with genes selected from differentially expressed UPSs may reject some candidates which have outstanding prognostic value but without differentially expressed significance. Considering this fact, we assume that a better prognostic predicting model may be constructed with these 391 prognosis-related genes recognized above without considering whether there are differentially expressed significances or not. With the standard log-rank p < 0.05 and | log2 hazard rate (HR) | > 1, we get 69 most prognosis-related genes with the help of GEPIA (**Figure 6B**). Our new prognostic model was formed with 28 genes of these 69 most prognosis-related UPSs by Cox regression analysis, we named it “**Model 2**”, shown in **Table 3**, and patients were classified into the high-risk group (n = 265) and the low-risk group (n = 265), survival analysis show that the survival rate of 1, 3, 5 years in low-risk group are about 96.7%, 89.8%, 83.9%, while in the high-risk group, the survival rate of 1, 3, 5 years are significantly reduced to only about 81.8%, 60.1%, 37.8%. The difference of survival rate is more obvious between two groups comparing with Model 1. We also visualized the relationship between risk score and patients' survival state as what we had done with Model 1, Cox regression analysis was also made in Model 2 (**Figure 8**).

### Estimation and external validation of Model 1 and Model 2

**Figure 9:**
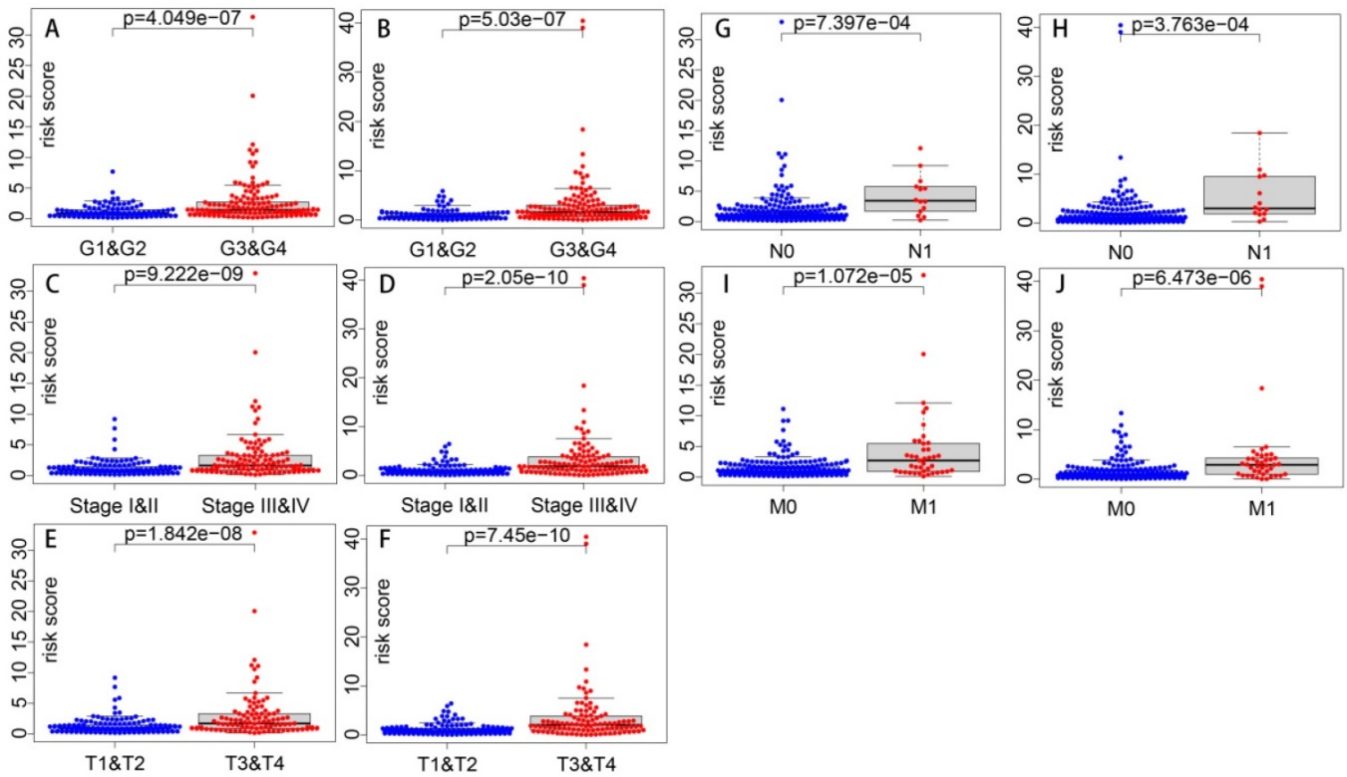
** Correlations of risk score with clinical parameters with statistical significance.** Correlations of risk score with grade in Model 1 (A) and Model 2 (B). Correlations of risk score with stage in Model 1 (C) and Model 2 (D). Correlations of risk score with T stage in Model 1 (E) and Model 2 (F). Correlations of risk score with N stage in Model 1 (G) and Model 2 (H). Correlations of risk score with M stage in Model 1 (I) and Model 2 (J).

Correlations of the risk score with clinical parameters were shown in **Table 4**, and correlations with statistical significance were shown in **Figure 9**. Results show that the risk score is strongly correlated with tumor grade, tumor stage, T stage, N stage and M stage. And the higher the risk score is, the higher the tumor grade and the later the tumor stage is likely to be.

**Figure 10:**
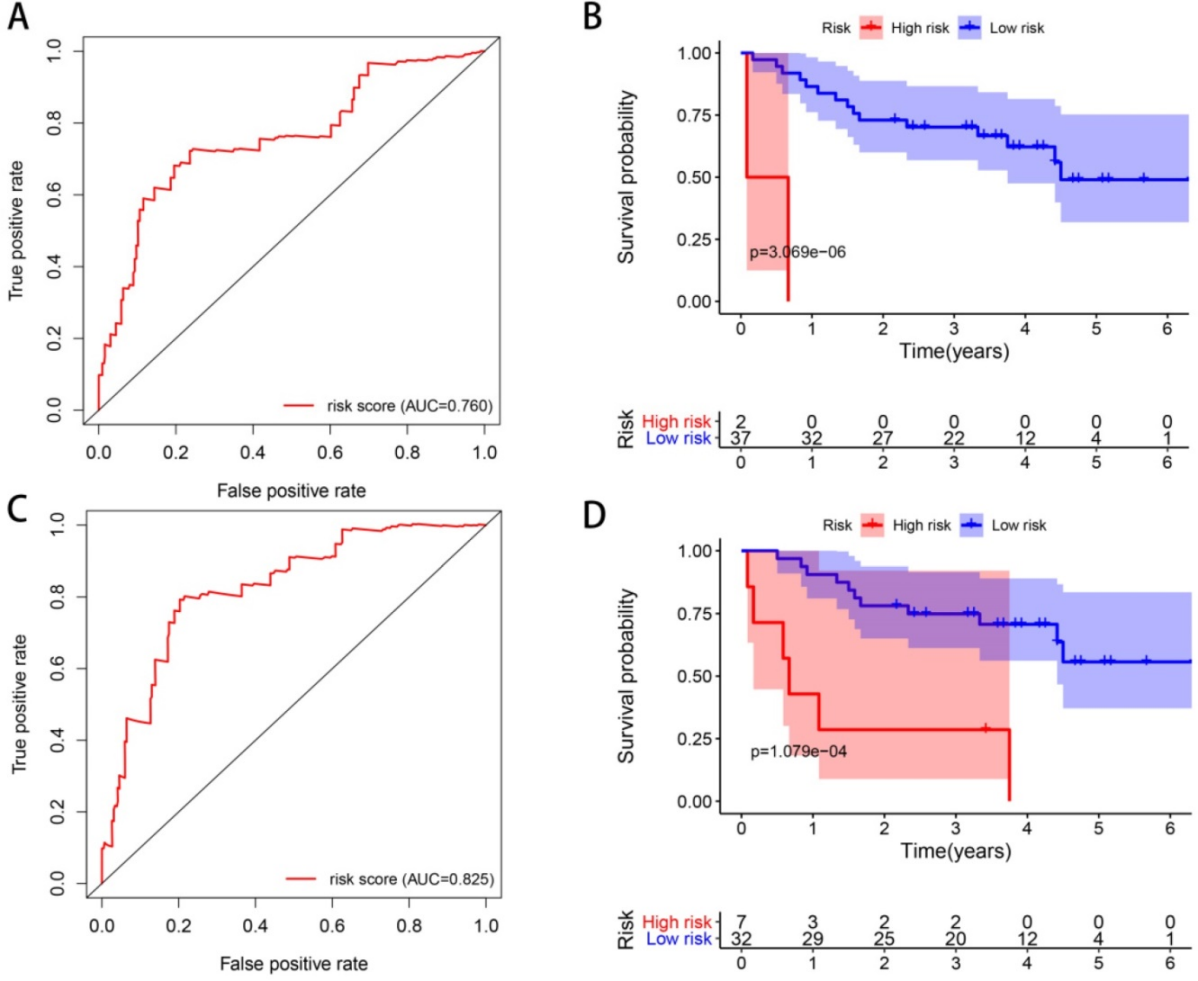
** ROC curves and external validations of Model 1 and Model 2.** ROC curves of Model 1 (A) and Model 2 (C). Kaplan-Meier curves of OS in the high- and low-risk groups stratified by the median risk score of Model 1 (B) and Model 2 (D) in GSE29609 cohort.

ROC curves were constructed to determine the predictive accuracy of the two different models. The area under the curve (AUC) for OS was 0.760 in Model 1, and 0.825 in Model 2, indicating both of them had good predictive accuracy, and Model 2 may be better (**Figure 10**). In order to further validate the prognostic predicting ability of our models, we downloaded expression and clinical data of 39 ccRCC patients in GSE29609 from GEO database, and calculated their risk scores with the formulas of Model 1 and Model 2, respectively. Patients in GSE29609 were classified into high and low risk groups with the median risk score of Model 1 and Model 2, respectively. As Kaplan-Meier curves in **Figure 10** show, the p values are as small as 3.069e-06 and 1.079e-04, indicating that our models remain effectively in this external cohort. All in all, the success of construction and validation of prognostic model with UPSs by two different approaches has strengthened our belief that the ubiquitin proteasome system (UPS) is of great importance in ccRCC, and models made with UPSs could predict the prognosis of patients effectively.

## Discussion

As early as 1991, Kanayama had explored the changes in expressions of ubiquitin and proteasome genes in renal cancer cells by Northern blot as well as immunochemical analysis, and they drew a conclusion that the ubiquitin and proteasome system should play a role in the renal cancer 36. In the past few decades, researchers have made great efforts to uncover the underlying mechanism of the UPS in the development of ccRCC 37-41. It is indisputable that we have made tremendous achievements in this field, however, on the other hand, it is unanimously agreed that there remain enormous appealing mechanisms waiting us to uncover. At the same time, there are some attempts trying to translate experimental findings of ccRCC into clinical applications. For instance, some UPS components are identified as pharmaceutical target to inhibit. Given bortezomib as an example, which is a proteasome inhibitor, the clinical trials have been done, but results are not very satisfactory 42, 43. There is no doubt that there is still a long way to go. Our results suggest there are more upregulated UPSs in tumor and UPS related pathways are highly enriched, while prognosis analysis implies there are more UPSs whose high expression followed with better survival. This seems paradoxical and indicates us UPS is definitively complicated and we should keep open-minded with UPS in searching potential targets and solutions 44, 45. Our differentially expressed analysis has identified some novel UPSs, for instance, RNF150, TRIM40, IRF2BPL, AREL1, NEURL3, FBXL6, LRRC41, KLHL17, RFPL4A, USP41, RNF149, PSMA8, CORO7, TRIM73, TRIM74, TRIML1, TRIML2, RNF113B and MARCH4. These UPSs have not been intensively studied and should be worth of further research.

When it comes to the prognostic value of UPS in ccRCC, there are researches focusing on specific UPS related genes, such as USP2 21 and SMURF1 46. But there are less systematically estimations of the whole ubiquitin proteasome system. With the development of the high-throughput sequencing and emergence of bioinformatic methods, such an exploration is practicable as well as attractive. Our study initially was aimed to draw a vivid picture of UPS in ccRCC from a bioinformatic perspective. Firstly, we conducted bioinformatic analysis of differential expression, functional enrichment and constructed Model 1 in a traditional way. 18 UPSs were involved in this prognostic predicting model. Some genes in Model 1 have been extensively studied such as CDC20 and ZNRF3. CDC20 was found to promote tumor cell migration and invasion for it was involved in the degradation of SMAR1, and SMAR1 is a tumor suppressor 47. The coefficient of CDC20 in our model is positive which means the higher CDC20 expression is, the higher the risk score will be, which is consistent with the previous researches. As for ZNRF3, a higher expression of ZNRF3 was found to be related with a better survival in colorectal carcinoma 48, while its role in ccRCC has not been elucidated. As its coefficient in our model is not only positive but also large, ZNRF3 may also play an important role in ccRCC.

With the help of GEPIA, we made a comprehensive analysis of the prognostic value of all 676 UPSs. Results show that 391 of them, which accounts for 57.84%, have significant difference between two groups separated by the expression value. It is so amazing to find such a big proportion of UPSs is strongly related with prognosis. This may at least suggest that there is an extensive dysregulation of UPS in ccRCC tissues. Additionally, we found many UPSs also have a strong relationship with tumor stage. These results indicate that the prognosis difference of UPSs may be partially explained by tumor stage, and some UPSs indeed have a good prediction of tumor stage. Furtherly, with the 69 most prognosis-related genes found in GEPIA, we constructed another prognostic model which was named Model 2. Model 2 is more exciting for it has a better predicting ability and with AUC up to 0.825. Model 2 is not only a better model maybe useful for further clinical diagnosis, but also strong evidence implying that the dysregulation of UPS must have something to do in ccRCC. Recently, research suggests that ccRCC is a metabolic disease with metabolic reprogramming covering a wide range of biological processes, such as fatty acid metabolism, aerobic glycolysis and amino acid metabolism 49. As a key pathway for protein degradation, UPS is also reported to be an essential modulator of cancer metabolism 50. Obviously, there requires more studies and efforts focusing on this field to develop novel diagnostic and therapeutic methods.

Finally, we estimated the relationship of the risk score with clinical parameters, and risk score was shown to have positive correlations with traditional parameters that can indicate the tumor malignancy. And further external validation with GSE29609 cohort successfully showed similar prognostic values of our models. However, our research also has some limitations. Firstly, our models seem to be a little complex for there are many genes involved in them. Secondly, further experiments *in vivo* and *in vitro* are still needed to validate the diagnostic and therapeutic value of these genes and our models. Additionally, there are some good prognosis models for ccRCC already 51-55. However, our models are very different from each other for the focus of our researches varies, and all signatures are treasures for they have enhanced our understanding of ccRCC. And in this study, we mainly focus on the ubiquitin proteasome system. If the small steps of our exploration do benefit patients suffering from ccRCC, we will feel gratified.

## Conclusion

In conclusion, we made a comprehensive exploration of the prognostic role of UPS in ccRCC from a bioinformatic perspective. UPS is dysregulated in ccRCC. UPS related genes have significant prognostic value in ccRCC. Models constructed with UPSs are effective and applicable. An abnormal ubiquitin proteasome system should play an important role in ccRCC. The ubiquitin proteasome system is a promising target for ccRCC and deserves further study.

## Supplementary Material

Supplementary table.

## Figures and Tables

**Table 1:** Clinical parameters of patients in TCGA-KIRC cohorts

Parameters	Cases	Proportion (%)
**Age (y)**		
≤65	352	65.55
>65	185	34.45
**Gender**		
Male	346	64.43
Female	191	35.57
**Stage**		
I	269	50.37
II	57	10.67
III	125	23.41
IV	83	15.55
**T stage**		
T1	275	51.21
T2	69	12.85
T3	182	33.89
T4	11	2.05
**N stage**		
N0	240	93.39
N1	17	6.61
**M stage**		
M0	426	84.36
M1	79	15.64
**Grade**		
G1	14	2.65
G2	230	43.48
G3	207	39.13
G4	78	14.74

**Table 2:** Differentially expressed UPSs in tumor samples comparing with normal samples

Dysregulation	Differentially expressed UPSs
Downregulated	ASB15, HECW1, RNF150, USP44, FBXO2, RNF43, DTX1, KLHL13, MARCH10, CBLC, UCHL1, USP2, TRIM2, TRIM40, CADPS2, RHOBTB3, LNX1, IRF2BPL, AMFR, USP46, RNF182, ZNRF3, PELI2, KLHL21, AREL1, PDZRN3, DCAF11, RNF152, NEDD4L, BRCC3, ASB9, NEURL3
Upregulated	MDM4, KCTD13, TRAF2, PCGF1, TRAF3IP2, WSB1, TRIM31, UBE2L6, FBXL6, LRRC41, CHFR, KLHL17, NEURL1B, RBCK1, TRIM59, SH3RF3, RACK1, PML, SPSB1, RFPL4A, TRIM36, DTX2, TRIM15, TNFAIP3, USP41, RNF149, PRC1, BCL6B, PSMB10, TRIM22, PSMA8, ASB2, MARCH3, HECW2, MYC, CORO7, FBXO17, PSMB8, MARCH1, CDC20, CDCA3, TRIM46, DDB2, TRIM73, DTL, TRIM74, PSMB9, BIRC3, TRIML1, STAMBPL1, UHRF1, TRIML2, RNF113B, MARCH4, UBE2C, TRIM9, RASD2, IFNG, BIRC7

**Table 3:** Risk score formula of Model 1 and Model 2

Model 1		Model 2	
Genes	Coefficient	Genes	Coefficient
CDCA3	0.647733	CDCA3	0.813077
CADPS2	-0.34575	RHOBTB1	-0.24355
USP2	-0.16609	FBXL5	-0.52466
PSMB10	-0.32733	PJA2	0.480908
CDC20	0.572892	FBXO7	0.57878
DTX1	0.257956	KLHL9	-1.31442
SPSB1	-0.19703	RNF20	-1.46314
PELI2	-0.40024	CDC20	0.596902
RNF149	0.506406	SOCS6	-0.57623
UBE2C	-0.43403	UBE2D3	-1.08593
CHFR	0.886869	OTUD7A	-1.42335
RNF43	-0.44588	PDE4D	-0.61161
BCL6B	0.293386	PELI2	-1.01604
HECW2	-0.49501	TOPORS	0.660042
RASD2	0.34123	PEX12	0.770969
MARCH4	-1.06908	UBE2C	-0.62467
ZNRF3	3.390968	TRIM2	-0.5344
TRIM15	-0.14047	EP300	-1.08857
		BFAR	-0.61988
		RAG1	-1.53854
		RNF38	0.751749
		MYLIP	0.50646
		KLHL8	1.201221
		TBL1X	0.309252
		TRAF6	0.762172
		FBXL4	0.962905
		FBXW2	1.622785
		ZNRF3	4.939872

**Table 4:** Correlations of risk score with clinical parameters (p value)

Clinical parameters	Models	
	Model 1	Model 2
Age	0.208	0.262
Gender	0.105	0.23
Grade	4.049e-07	5.03e-07
Stage	9.222e-09	2.05e-10
T	1.842e-08	7.45e-10
N	7.397e-04	3.763e-04
M	1.072e-05	6.473e-06

## Abbreviations

AUC: area under the curve
ccRCC: clear cell renal cell carcinoma
DUBs: deubiquitinating enzymes
E1s: ubiquitin activating enzymes
E2s: ubiquitin conjugating enzymes
E3s: ubiquitin ligases
FC: fold change
FDR: false discovery rate
GO: gene ontology
GSEA: Gene Set Enrichment Analysis
HIF: hypoxia-inducible-factor
HR: hazard rate
KEGG: Kyoto Encyclopedia of Genes and Genomes
KIRC: Kidney renal clear cell carcinoma
OS: overall survival
RCC: renal cell carcinoma
ROC: receiver operating characteristic
TCGA: the cancer genome atlas
UPS: ubiquitin proteasome system
UPSs: UPS-related genes
VHL: Von-Hippel Lindau
