# Supplementary material for: Prognostic Role of the Ubiquitin Proteasome System in Clear Cell Renal Cell Carcinoma: A Bioinformatic Perspective
Source: J Cancer. 2021 May 13;12(14):4134–47. doi: 10.7150/jca.53760 (PMC8176417; doi:10.7150/jca.53760)
Supplement: Supplementary file 1 — Supplementary table. [file jcav12p4134s1.pdf]

Table S1. Set of 676 ubiquitin proteasome system related genes

Categories	Genes
Ubiquitin	RPS27A, UBA52, UBB, UBC
Ubiquitin activating enzymes (E1s)	UBA1, UBA6
Ubiquitin conjugating enzymes (E2s)	UBE2A, UBE2B, UBE2C, UBE2D1, UBE2D2, UBE2D3, UBE2D4, UBE2E1, UBE2E2, UBE2E3, UBE2G1, UBE2G2, UBE2H, UBE2J1, UBE2J2, UBE2K, UBE2L3, UBE2N, UBe2NL, UBE2O, UBE2Q1, UBE2Q2, UBe2QL, UBE2R1, UBE2R2, UBE2S, UBE2T, UBE2U, UBE2V1, UBE2V2, UBE2W, BIRC6, UBE2F, UBE2I, UBE2L6, UBE2M, UBE2Z, ATG10, ATG3
Ubiquitin ligases (E3s)	HECTD4, G2E3, HACE1, HECTD1, HECTD2, HECW1, HERC2, HERC4, HERC6, HUWE1, ITCH, NEDD4, SMURF2, TRIP12, UBE3B, WWP1, WWP2, HERC5, UBE3A, UBE3C, SMURF1, NEDD4L, HECTD3, HERC1, UBE3D, HERC3, TRIP12, HECW2, UBR5, ZNF645, SYVN1, RNF168, BIRC2, CBLB, CNOT4, MARCH7, MSL2, MYLIP, PDZRN3, RAD18, RNF111, RNF125, RNF128, RNF31, RNF6, SIAH2, TRIM24, TRIM28, TRIM5, XIAP, ZNRF2, AMFR, PIAS2, BARD1, BIRC3, BIRC7, BMI1, BRAP, BRCA1, CBL, CBLC, CBLL1, CCNB1IP1, CHFR, DTX1, DTX2, DTX3, DTX3L, RNF7, FANCL, HLTF, RBCK1, TRIM32, LNX1, MARCH1, MARCH11, MARCH2, MARCH3, MARCH4, MARCH6, MARCH8, MARCH9, MDM2, MGRN1, MID1, MID2, MKRN1, MNAT1, MUL1, MYCBP2, NHLRC1, PARK2, PEX10, PEX12, PEX2, PIAS1, PIAS3, PIAS4, PML, RAG1, RBBP6, RBX1, RC3H1, RCHY1, RFFL, RFPL4A, RFWD3, RING1, RNF181, RNF103, RNF11, RNF114, RNF115, RNF121, RNF122, RNF126, RNF13, RNF130, RNF138, RNF139, RNF144B, RNF146, RNF152, RNF167, RNF170, RNF180, RNF182, RNF187, RNF19A, RNF2, RNF213, RNF217, RNF220, RNF34, RNF38, RNF4, RNF40, RNF41, RNF5, RNF8, SH3RF1, SHPRH, SIAH1, TRIM51, TOPORS, TRAF2, TRAF5, TRAF6, TRIM11, TRIM13, TRIM15, TRIM17, TRIM2, TRIM21, TRIM23, TRIM25, TRIM26, TRIM27, TRIM3, TRIM31, TRIM35, TRIM36, TRIM37, TRIM38, TRIM39, TRIM4, TRIM40, TRIM41, TRIM43, TRIM45, TRIM47, TRIM50, TRIM54, TRIM56, TRIM59, TRIM6, TRIM62, TRIM63, TRIM68, TRIM73, TRIM74, TRIM8, TRIM9, TTC3, UHRF1, UHRF2, VPS18, ZNRF1, ZNRF4, BFAR, DZIP3, MDM4, NFX1, PCGF1, PJA1, PJA2, RNF123, RNF133, RNF135, RNF20, RNF25, ZNRF3, LRSAM1, NSMCE2, ZMIZ1, ZMIZ2, NSMCE1, PELI2, PELI1, PELI3, ARIH1, RNF14, RNF19B, RNF216, TRIM46, ZSWIM2, ARIH2, MIB1, TRIM22, MARCH5, RLIM, OSTM1, RNF185, DTX4, TRIM71, NEURL1B, PCGF2, RNF113A, RNF113B, RNF150, RNF186, RNF24, RNF43, RNFT1, TRIM34, TRIM65, VPS41, TRAF3, SH3RF3, LITAF, RNF26, IRF2BP1, LTN1, TRAIP, MARCH10, MIB2, UBR1, UBR2, TRIM33, UBOX5, CUL9, RFWD2, TRAF7, STUB1, UBE4A, UBE4B, NOSIP, TRAF3IP2, PPIL2, PRPF19, UBR4, HDAC4, RASD2, UFL1, EP300, ZFP91, E4F1, RABGEF1, KAT2B, KCMF1, CBX4, DCUN1D1, DCUN1D3, DCUN1D2, PDLIM2, MAP3K1, AIRE, RWDD3, FUS, RANBP2, STC1,

	MALT1, AURKA, SART1, CREBBP, CRBN, IRF2BPL, CADPS2, RMND5B, KDM2B, TNFAIP3, UCHL1, ANAPC2, CUL4A, CUL5, CUL3, CUL7, CUL1, CUL2, CUL4B, FBXO3, CCNF, FBXO44, FBXL15, FBXL3, FBXO11, FBXO17, FBXO18, FBXO2, FBXO22, FBXO25, FBXO27, FBXO31, FBXO33, FBXO4, FBXO45, FBXO6, FBXO7, FBXO8, FBXW10, FBXO32, FBXO42, FBXL2, FBXL6, FBXL12, FBXW8, FBXL14, FBXL5, FBXL4, SKP2, CDCA3, ZC3HC1, FBXO40, FBXL7, FBXL19, FBXW5, FBXW2, BTRC, FBXW11, FBXW7, TBL1X, TBL1XR1, VHL, MED8, LRRC41, TMF1, ASB1, ASB3, ASB6, ASB8, ASB9, NEURL2, SOCS5, SOCS6, SOCS7, SPSB1, ASB2, ASB4, RAB40C, ASB12, ASB7, SOCS1, SOCS2, SOCS3, SOCS4, SPSB4, CISH, FEM1B, LRR1, ASB15, SPSB2, WSB2, WSB1, CORO7, TCEB1, TCEB2, KLHL42, KLHL12, KLHL17, KLHL8, SPOP, KLHL13, KLHL10, KLHL2, KLHL20, KLHL22, KLHL7, KLHL21, RCBTB1, KBTBD13, KEAP1, KLHL9, KLHL41, GAN, KCTD11, KCTD13, KCTD21, KCTD6, TNFAIP1, ABTB1, BCL6B, RHOBTB1, RHOBTB2, KCTD5, ZBTB18, NACC1, RHOBTB3, VPRBP, DDB2, ERCC8, DTL, GNB2L1, DCAF12, DCAF7, GRWD1, PWP1, WDR53, DCAF4, DCAF5, KATNB1, NUP43, RBBP4, RBBP5, TLE1, TLE2, WDR82, SMU1, WDR26, ATG16L1, CIAO1, DCAF13, DCAF6, EED, GNB2, POC1B, RBBP7, SNRNP40, TLE3, WDR12, WDR5, WDR5B, WDR61, WDR76, DCAF10, DCAF8, WDTC1, PAFAH1B1, BRWD1, NLE1, PHIP, WDR59, AMBRA1, DCAF11, DDB1, TRPC4AP, AHR, HOXB4, RICTOR, DCAF15, DCAF16, DCAF17, ARNT, FZR1, CDC20, CDC27, ANAPC11, ANAPC4, ANAPC5, ANAPC7, ANAPC1, CDC23, ANAPC10, CDC16, CDC26, ANAPC13, AREL1, MAGEA6, MEX3C, MKRN2, MKRN3, MKRN4P, MYC, NEURL1, NEURL3, PDE4D, RNF144A, RNF149, RNF169, SH3RF2, TRIM69, TRIML1, TRIML2, UBE2E3, UBAC1, UBR3, UBR7, UNKL, PRC1, IRAK1, IRAK4
Deubiquitinating enzymes (DUBs)	BAP1, UCHL1, UCHL3, UCHL5, ATXN3, ATXN3L, JOSD1, JOSD2, TNFAIP3, ZA20D1, OTUD7A, OTUD4, PARP11, OTUD6A, YOD1, OTUD6B, OTUD5, OTUB1, OTUB2, OTUD1, ZRANB1, VCPIP1, STAMBP, STAMBPL1, BRCC3, COPS5, COPS6, EIF3S3, EIF3S5, Q7Z3G8, PSMD14, PRPF8 PSMD7, USP1, USP2, USP3, USP4, USP5, USP6, USP7, USP8, USP9X, USP9Y, USP10, USP11, USP12, USP13, USP14, USP15, USP16, USP18, USP19, USP20, USP21, USP22, USP24, USP25, USP26, USP28, USP29, USP30, USP31, USP32, USP33, USP34, USP35, USP36, USP37, USP38, USP39, USP40, USP41, USP42, USP44, USP45, USP46, USP47, USP48, USP49, USP50, USP51, USP52, USP54, USP43, CYLD
Proteasome	IFNG, POMP, PSMA1, PSMA2, PSMA3, PSMA4, PSMA5, PSMA6, PSMA6P4, PSMA7, PSMA8, PSMB1, PSMB10, PSMB11, PSMB2, PSMB3, PSMB4, PSMB5, PSMB6, PSMB7, PSMB8, PSMB9, PSMC1, PSMC1P4, PSMC2, PSMC3, PSMC4, PSMC5, PSMC6, PSMD1, PSMD11, PSMD12, PSMD13, PSMD14, PSMD2, PSMD3, PSMD4, PSMD6, PSMD7, PSMD8, PSME1, PSME2, PSME3, PSME4, PSMF1, SEM1

Table S2. Functional enrichment of the differentially expressed UPSs

Category	ID	Description	pvalue	geneID
GO-BP	GO:0000209	protein polyubiquitination	2.07E-23	WSB1/RBCK1/TNFAIP3/PSMB10/DTL/PSMB8/PSMB9/HECW1/AREL1/PSMA8/TRAF2/NEDD4L/DDB2/TRIM36/SPSB1/PELI2/UBE2C/TRIM2/CHFR/AMFR/FBXO17/HECW2/FBXO2/RNF152
GO-BP	GO:0043161	proteasome-mediated ubiquitin-dependent protein catabolic process	1.34E-21	DCAF11/RBCK1/PSMB10/RACK1/PML/PSMB8/PSMB9/HECW1/CDC20/AREL1/KCTD13/PSMA8/TRIM9/NEDD4L/SPSB1/UCHL1/UBE2C/TRIM2/CHFR/AMFR/USP44/FBXO17/FBXL6/HECW2/FBXO2
GO-BP	GO:0010498	proteasomal protein catabolic process	3.14E-20	DCAF11/RBCK1/PSMB10/RACK1/PML/PSMB8/PSMB9/HECW1/CDC20/AREL1/KCTD13/PSMA8/TRIM9/NEDD4L/SPSB1/UCHL1/UBE2C/TRIM2/CHFR/AMFR/USP44/FBXO17/FBXL6/HECW2/FBXO2
GO-BP	GO:0016579	protein deubiquitination	4.03E-16	USP2/TNFAIP3/PSMB10/PSMB8/PSMB9/CDC20/PSMA8/TRAF2/BRCC3/USP46/BIRC3/DDB2/USP41/UCHL1/USP44/MDM4/MYC/STAMBPL1
GO-BP	GO:0070646	protein modification by small protein removal	1.06E-15	USP2/TNFAIP3/PSMB10/PSMB8/PSMB9/CDC20/PSMA8/TRAF2/BRCC3/USP46/BIRC3/DDB2/USP41/UCHL1/USP44/MDM4/MYC/STAMBPL1
GO-BP	GO:0043687	post-translational protein modification	3.99E-13	DCAF11/ASB9/WSB1/PSMB10/LRRC41/ASB2/DTL/PSMB8/PSMB9/PSMA8/KLHL21/DDB2/SPSB1/KLHL13/FBXO17/FBXO2/ASB15
GO-BP	GO:0042176	regulation of protein catabolic process	1.47E-08	ASB9/TNFAIP3/RACK1/PML/DTL/IFNG/HECW1/AREL1/NEDD4L/CHFR/MDM4/HECW2/TRIM40
GO-BP	GO:0045732	positive regulation of protein catabolic	4.18E-08	ASB9/TNFAIP3/RACK1/DTL/IFNG/HECW1/AREL1/NEDD4L/CHFR/HECW2

		process		
GO-BP	GO:0033209	tumor necrosis factor-mediated signaling pathway	6.12E-08	RBCK1/TNFAIP3/PSMB10/RACK1/PSMB8/PSMB9/PSMA8/TRAF2/BIRC3
GO-BP	GO:0031146	SCF-dependent proteasomal ubiquitin-dependent protein catabolic process	2.32E-07	PSMB10/PSMB8/PSMB9/PSMA8/FBXO17/FBXL6/FBXO2
GO-CC	GO:0000151	ubiquitin ligase complex	4.29E-18	DCAF11/RBCK1/ASB2/DTL/CDC20/KCTD13/UBE2L6/KLHL21/TRAF2/BRCC3/DDB2/SPSB1/UBE2C/PCGF1/AMFR/KLHL13/FBXO17/FBXL6/FBXO2
GO-CC	GO:0031461	cullin-RING ubiquitin ligase complex	2.35E-12	DCAF11/DTL/CDC20/KCTD13/KLHL21/DDB2/SPSB1/UBE2C/KLHL13/FBXO17/FBXL6/FBXO2
GO-CC	GO:0005839	proteasome core complex	1.28E-06	PSMB10/PSMB8/PSMB9/PSMA8
GO-CC	GO:0019774	proteasome core complex, beta-subunit complex	1.12E-05	PSMB10/PSMB8/PSMB9
GO-CC	GO:0000152	nuclear ubiquitin ligase complex	3.03E-05	CDC20/BRCC3/UBE2C/PCGF1
G	GO:	proteaso	0.0	PSMB10/PSMB8/PSMB9/PSMA8

O-CC	0000502	me complex	00137331	
GO-CC	GO:0019005	SCF ubiquitin ligase complex	0.000146025	SPSB1/FBXO17/FBXL6/FBXO2
GO-CC	GO:1905369	endopeptidase complex	0.000146025	PSMB10/PSMB8/PSMB9/PSMA8
GO-CC	GO:0080008	Cul4-RING E3 ubiquitin ligase complex	0.000345686	DCAF11/DTL/DDB2
GO-CC	GO:0031463	Cul3-RING ubiquitin ligase complex	0.000486437	KCTD13/KLHL21/KLHL13
GO-MF	GO:0004842	ubiquitin-protein transferase activity	1.46E-45	BIRC7/RBCK1/TNFAIP3/NEURL3/LNX1/ASB2/DTL/HECW1/NEURL1B/AREL1/KCTD13/RNF182/UBE2L6/KLHL21/TRAF2/TRIM9/BIRC3/NEDD4L/DDB2/TRIM36/PELI2/RNF149/UBE2C/RNF150/TRIM2/CHFR/TRIM59/AMFR/IRF2BPL/CBLC/KLHL13/RNF43/FBXO17/FBXL6/HECW2/UHRF1/PDZRN3/FBXO2/RNF152/SH3RF3/ZNRF3
GO-MF	GO:0019787	ubiquitin-like protein transferase activity	2.12E-44	BIRC7/RBCK1/TNFAIP3/NEURL3/LNX1/ASB2/DTL/HECW1/NEURL1B/AREL1/KCTD13/RNF182/UBE2L6/KLHL21/TRAF2/TRIM9/BIRC3/NEDD4L/DDB2/TRIM36/PELI2/RNF149/UBE2C/RNF150/TRIM2/CHFR/TRIM59/AMFR/IRF2BPL/CBLC/KLHL13/RNF43/FBXO17/FBXL6/HECW2/UHRF1/PDZRN3/FBXO2/RNF152/SH3RF3/ZNRF3
GO-MF	GO:0061630	ubiquitin protein ligase activity	6.73E-31	BIRC7/NEURL3/ASB2/HECW1/NEURL1B/AREL1/BIRC3/NEDD4L/PELI2/RNF149/RNF150/TRIM2/CHFR/TRIM59/AMFR/IRF2BPL/CBLC/RNF43/FBXO17/FBXL6/HECW2/UHRF1/PDZRN3/FBXO2/RNF152/SH3RF3/ZNRF3
GO-	GO:006165	ubiquitin-like protein	2.05E-	BIRC7/NEURL3/ASB2/HECW1/NEURL1B/AREL1/BIRC3/NEDD4L/PELI2/RNF149/RNF150/TRIM2/CHFR/TRIM59/AMFR/IRF2BPL/CBLC/RNF43/FBXO17/FBXL6/HECW2/UHRF1/PDZR

MF	9	ligase activity	30	N3/FBXO2/RNF152/SH3RF3/ZNRF3
GO-MF	GO:0004843	thiol-dependent ubiquitin-specific protease activity	4.57E-08	USP2/TNFAIP3/BRCC3/USP46/USP41/UCHL1/USP44/STAMBPL1
GO-MF	GO:0036459	thiol-dependent ubiquitinyl hydrolase activity	8.09E-08	USP2/TNFAIP3/BRCC3/USP46/USP41/UCHL1/USP44/STAMBPL1
GO-MF	GO:0101005	ubiquitinyl hydrolase activity	8.09E-08	USP2/TNFAIP3/BRCC3/USP46/USP41/UCHL1/USP44/STAMBPL1
GO-MF	GO:0019783	ubiquitin-like protein-specific protease activity	1.46E-07	USP2/TNFAIP3/BRCC3/USP46/USP41/UCHL1/USP44/STAMBPL1
GO-MF	GO:0004298	threonine-type endopeptidase activity	2.92E-06	PSMB10/PSMB8/PSMB9/PSMA8
GO-MF	GO:0070003	threonine-type peptidase activity	2.92E-06	PSMB10/PSMB8/PSMB9/PSMA8
KEGG	hsa04120	Ubiquitin mediated proteolysis	2.85E-12	BIRC7/PML/CDC20/UBE2L6/BIRC3/NEDD4L/DDB2/UBE2C/CBLC/KLHL13/FBXO2
KEGG	hsa03050	Proteasome	8.82E-07	PSMB10/IFNG/PSMB8/PSMB9/PSMA8

KEGG	hsa05222	Small cell lung cancer	2.78E-05	BIRC7/TRAF2/BIRC3/DDB2/MYC
KEGG	hsa04217	Necroptosis	0.000372211	RBCK1/TNFAIP3/IFNG/TRAF2/BIRC3
KEGG	hsa04657	IL-17 signaling pathway	0.000491297	TNFAIP3/IFNG/TRAF2/TRAF3IP2
KEGG	hsa04621	NOD-like receptor signaling pathway	0.000674031	RBCK1/TNFAIP3/TRAF2/BRCC3/BIRC3
